# Spatial analysis of contaminated areas in the Centro de Referência em Saúde do Trabalhador of Osasco region (SP), 2017

**DOI:** 10.47626/1679-4435-2022-974

**Published:** 2024-08-05

**Authors:** Aparecido Batista de Almeida, Alessandra Cristina Guedes Pellini

**Affiliations:** 1 Centro de Vigilância Epidemiológica, São Paulo, SP, Brazil; 2 Faculdade de Saúde Pública, Universidade de São Paulo, São Paulo, SP, Brazil; 3 Universidade Nove de Julho, São Paulo, SP, Brazil; 4 Coordenação de Epidemiologia e Informação, São Paulo, SP, Brazil

**Keywords:** spatial analysis, service stations, contaminated areas, hydrocarbons, análise espacial, postos de serviço, áreas contaminadas, hidrocarbonetos

## Abstract

**Introduction:**

According to the Instituto Brasileiro de Geografia e Estatística (Brazilian Institute of Geography and Statistics), 33% of Brazilian municipalities have faced problems with areas of contaminated soil. According to Companhia Ambiental do Estado de São Paulo (State of São Paulo Environmental Company), contaminated areas are places where there is demonstrably pollution or contamination.

**Objective:**

To identify the areas and municipalities affected by contamination at fuel service stations in the Centro de Referência em Saúde do Trabalhador (Occupational Health Reference Center) of Osasco region in 2017.

**Methods:**

Descriptive ecological study, carried out in the Occupational Health Reference Center of Osasco region. Data on contaminated areas were extracted from State of São Paulo Environmental Company and digital maps from Brazilian Institute of Geography and Statistics. Spatial analysis was carried out of the contamination areas, according to their classification, as well as artesian wells and service stations, using Geographic Information System tools.

**Results:**

The highest concentrations of contaminated areas with confirmed risk are found in the municipality of Osasco and headquarters of Occupational Health Reference Center. In the analysis of the Kernel Ratio, the most compromised municipalities were Osasco, Barueri and Taboão da Serra.

**Conclusions:**

Municipalities with contaminated areas with confirmed risk also have water extraction from artesian wells, with potential risk of contamination of this water by benzene. Thus, it is necessary to improve inspection and surveillance of areas with environmental liabilities, such as service stations that do not have remediation, in addition to surveillance of exposed workers.

## Abstract (pt)

**Introdução:**

De acordo com o Instituto Brasileiro de Geografia e Estatística, 33% dos municípios brasileiros têm enfrentado problemas com áreas de solo contaminado. Segundo a Companhia Ambiental do Estado de São Paulo, as áreas contaminadas são locais onde há poluição ou contaminação por substâncias ou resíduos.

**Objetivo:**

Identificar as áreas e os municípios afetados por contaminação em postos de serviços de combustível na região do Centro de Referência em Saúde do Trabalhador de Osasco, no estado de São Paulo, em 2017.

**Métodos:**

Tratou-se de um estudo descritivo ecológico, realizado na área do Centro de Referência em Saúde do Trabalhador de Osasco. Os dados de áreas contaminadas foram extraídos da Companhia Ambiental do Estado de São Paulo, e os mapas digitais, do Instituto Brasileiro de Geografia e Estatística. Foi realizada análise espacial das áreas de contaminação segundo a sua classificação, bem como de poços artesianos e postos de combustível, utilizando ferramentas de Sistemas de Informações Geográficas.

**Resultados:**

As maiores concentrações de áreas contaminadas com risco confirmado se encontram no município de Osasco, sede do Centro de Referência em Saúde do Trabalhador. Na análise da razão de Kernel, os municípios mais comprometidos foram Osasco, Barueri e Taboão da Serra.

**Conclusões:**

Os municípios com áreas contaminadas com risco confirmado possuem extração de água de poços artesianos, com potencial risco de contaminação dessa água por benzeno. Assim, é necessário melhorar a fiscalização e a vigilância das áreas com passivo ambiental, como postos de gasolina.

## INTRODUCTION

Contaminated areas have a negative impact on environment and human beings. The need to manage these areas arose from the significant increase in major contamination events worldwide in the 20th century. Some examples are the Love Canal neighborhood in New York, where tons of industrial waste were found in backyards, basements, and residential plumbing in the 1970s, and the city of Cubatão, SP, Brazil, where atmospheric pollution caused by industrial production heavily affected the Atlantic Forest and killed several species of native flora in the early 1980s.^1^ Recently, a dam burst in Brumadinho, MG, and caused the death of several workers, local residents and animals, and dumped ore tailings into the Paraopeba River.

According to the Instituto Brasileiro de Geografia e Estatística (IBGE, Brazilian Institute of Geography and Statistics), 33% of Brazilian municipalities have had problems with contaminated soil, and the primary sources of contamination are the use of fertilizers and pesticides and the improper disposal of domestic sewage.^2^

Contaminated areas are sites where pollution or contamination has been proven to be caused by the introduction of any substances or waste, whether accumulated, stored, buried, or infiltrated either deliberately, accidentally, or even naturally.^3^ According to Brazilian legislation, contaminated areas are categorized based on the possibility of harm to humans, water, buildings, or ecosystems.^4^ These contaminants can be concentrated in the subsurface in different compartments of the environment: soil, sediments, rocks, materials used to fill land, and groundwater, besides walls, floors, and other building structures.^5,6^

In urban areas, one of the most significant sources of environmental contamination are fuel spills at service stations and refinery plants at storage and distribution sites, and also accidents involving fuel tankers. Fuel spills at service stations are mainly due to fuel storage system not being sealed, corrosion in the metal structures of tanks and pipes, and inadequate operating procedures. This type of contamination is a major cause for concern, since the various workers in this chain are exposed to hydrocarbons contained in fuels, which can spread over large areas, carried by groundwater, thus ruining the quality of this resource, the soil, and human health in general.^7,8^

Polycyclic aromatic hydrocarbons are lipophilic compounds and are therefore rapidly absorbed by all routes of exposure, whether through inhalation, orally, or dermally. They also have carcinogenic properties that are harmful to humans and animals, and are directly related to road traffic and industrial pollution.^9^

Benzene, a volatile, colorless, sweet-smelling compound widely used in the oil industry, is one of the hydrocarbons present in fuels and can be present in the air, soil, or water.^10,11^ Since 1982, the International Agency for Research on Cancer and the World Health Organization have both recognized benzene as a carcinogen, mainly for leukemia (a malignant disease of leukocytes). In 1994, Brazil Comissão Nacional Permanente do Benzeno (National Permanent Benzene Commission) was formed to define guidelines to restrict benzene from circulating in companies that produce, transport, store, use, or handle the product in a liquid mixture at a concentration of at least 1% for workers exposure.^11^

Acute (short-term) human exposure to benzene through inhalation can cause drowsiness, dizziness, headache, eye pain, irritation of the skin and respiratory tract, and loss of consciousness when at high levels. Exposure through chronic (longterm) inhalation of benzene can cause various blood disorders, including a reduced number of red blood cells and aplastic anemia, especially in occupational settings, such as service stations workers.^11^

The advent of the Unified Health System (SUS), introduced in the 1988 Brazilian Constitution, represented a legal and juridical milestone towards social security in Brazil, based on a series of integrated actions at the 3 levels of government and the participation of the civil society, in order to guarantee the rights to health, social security, and social assistance.^12^ In 1990, Organic Health Law No. 8.080 set forth principles and guidelines: universal right to health; decentralization, with a single directorate in each government level; and comprehensive health care and popular participation, with a view to social control. Similarly, occupational health has also been following these transformations with the SUS after more than 30 years, jointly cooperating with sanitary, epidemiological, and environmental surveillance to address occupational health-related processes with contemporary monopolies and oligopolies.^12^

In July 2018, the 307th ordinary meeting of the National Health Council passed Resolution No. 588, which introduced the Política Nacional de Vigilância em Saúde (PNVS, National Health Surveillance Policy). In Article 3, it states: “the PNVS comprises the articulation of knowledge, processes, and practices related to epidemiological surveillance, environmental health surveillance, occupational health surveillance, and health surveillance. This policy is in line with the set of health policies within the SUS, considering the cross-cutting nature of health surveillance actions on defining health-disease process.”

One of the definitions of the PNVS in Article 6 is: “XI - Occupational health surveillance: a set of actions aimed at promoting health, preventing morbidity and mortality, and reducing risks and vulnerabilities among the labor force, through the integration of actions that intervene in diseases and illnesses and their determinants that result from development models, production processes, and work.”

Article 8 presents the following guideline: “integrate work practices and processes of epidemiological, sanitary, environmental, and occupational health surveillance and public health laboratories, preserving their specificities, sharing knowledge and technologies, fostering multiprofessional and interdisciplinary work.”

The Centro de Referência em Saúde do Trabalhador (CEREST, Occupational Health Reference Center) in Osasco was chosen for this study in view of its importance to the Região Metropolitana de São Paulo (RMSP, São Paulo Metropolitan Region), since it covers two large regions: the Mananciais and the Rota dos Bandeirantes, with a population density of around 3 million inhabitants and 115,221 companies, a significant number with great economic potential. It also has important access routes, such as the Rodoanel, Rodovia dos Bandeirantes, Rodovia dos Imigrantes, Rodovia Ayrton Senna, and Rodovia Fernão Dias.^13,14^

Geographic Information Systems (GIS) are a group of software applications that integrate data, equipment, and people, aiming at collecting, storing, retrieving, manipulating, visualizing, and analyzing data spatially referenced to a known coordinate system. They are also capable of providing spatial analysis of public health problems, and are extremely useful in assessing the relationship between these problems and environmental and socioeconomic variables. GIS are therefore used to understand or clarify facts and phenomena that occur in geographical space.^15^

Innovative possibilities for studying the health situation and its trends have been offered by the great advance in the development of technologies that analyze data in geographical space, providing a better understanding of socioeconomic and environmental factors, among other factors that determine the living conditions and health status of the population.

Spatial distribution of contaminated or potentially contaminated areas is scarce, and there is a need for greater technical knowledge on the part of occupational health professionals to analyze these areas so as to guide decision-making and implement innovative and effective public policies.

In view of this scenario, this study aimed to identify areas with proven contamination from service stations in the region where Osasco CEREST operated in 2017.

## METHODS

### STUDY AREA

The Osasco CEREST is part of the West and Southwest regions in the largest metropolitan region in São Paulo, Brazil. As of 2018, its population was estimated at 3,021,960 people, and it had a population density of 3,394.55 inhabitants per square kilometer in the West region and 775.83 inhabitants per square kilometer in the Southwest. In 2016, the gross domestic product was around BRL 187,673,469, making this area strategic for the RMSP. It also has a large highway network with important accesses to the city of São Paulo.^14^

The Osasco CEREST region has 15 municipalities, namely: Barueri, Carapicuíba, Itapevi, Jandira, Osasco, Pirapora do Bom Jesus, and Santana de Parnaíba in the West region; Cotia, Embu das Artes, Embu-Guaçu, Itapecerica da Serra, Juquitiba, São Lourenço da Serra, Taboão da Serra, and Vargem Grande Paulista in the Southwest region.^14^

### DATA SOURCES

This study used data from the 2017 Cadastro de Áreas Contaminadas (Registry of Contaminated Areas) of the Companhia Ambiental do Estado de São Paulo (CETESB, State of São Paulo Environmental Company) and the RMSP. This database provides a series of information on suspected and contaminated areas, distributed into categories according to the stage of the process of identification and restoration of contamination: contaminated area in the process of reuse (ACRu); contaminated area under investigation (ACI); contaminated area with confirmed risk (ACRi); contaminated area in the process of restoration (ACRe); area rehabilitated for declared use (AR); and area in the process of monitoring for closure (AME).

The digital cartographic bases of the study areas and the 2016 population were retrieved from the IBGE website. The databases of artesian wells in the study region were obtained from the Groundwater Information System (SIAGAS)^16^ of the Geological Survey of Brazil (SGB).

### DATA ANALYSIS

In 2017, 136 of the 226 contaminated areas in the Osasco CEREST region were geocoded (60.2%) using the MMQGIS plugin in the QGIS 2.2, Valmiera (2013). The remaining 90 contaminated areas (39.8%) were geocoded by active or manual search for the latitude and longitude geographic coordinates of these areas on the EasyMapMaker website (based on Google Earth), totaling 100% of the contaminated areas georeferenced. The following data from the Registry of Contaminated Areas was added to the attribute tables of the geocoded points: company name and main activity (industry, business, or fuel service station).

In order to spatially display and visualize the density of points aggregating all the contaminated areas, a Kernel estimate map (heat map) of the region was drawn up using the interpolation method in TerraView 4.2.2 (2013) and ArcGIS 10.1 (2012). This is an exploratory interpolation technique that generates a density surface for the visual identification of “hot areas.”^15^

Based on the relationship between Kernel estimate and population density, a “population at risk” surface was created, thus generating an estimate called the Kernel ratio.^15^ Thus, the variable “number of events” (contaminated points or areas) was related to the variable “population”, which is an attribute of the census tract layer. The IBGE 2016 population was used to estimate the Kernel ratio.

A map covering 600-meter radii around the centroid points of the contaminated areas (buffer dissove tool) in the QGIS 2.2 application was drawn up in order to infer the potential for groundwater contamination at service stations. The 600-meter radius was drawn up following Frank et al.^17^ findings so as to understand the impact of contaminated areas, in terms of slope, proximity to water resources, and the characteristics of land use and occupation.

SIAGAS^16^ surveyed artesian wells throughout the Osasco CEREST region registered as being used for human and animal consumption, leisure, and crop irrigation. This led to a list of contaminated groundwater areas in the region.

## RESULTS

**Figure 1:**
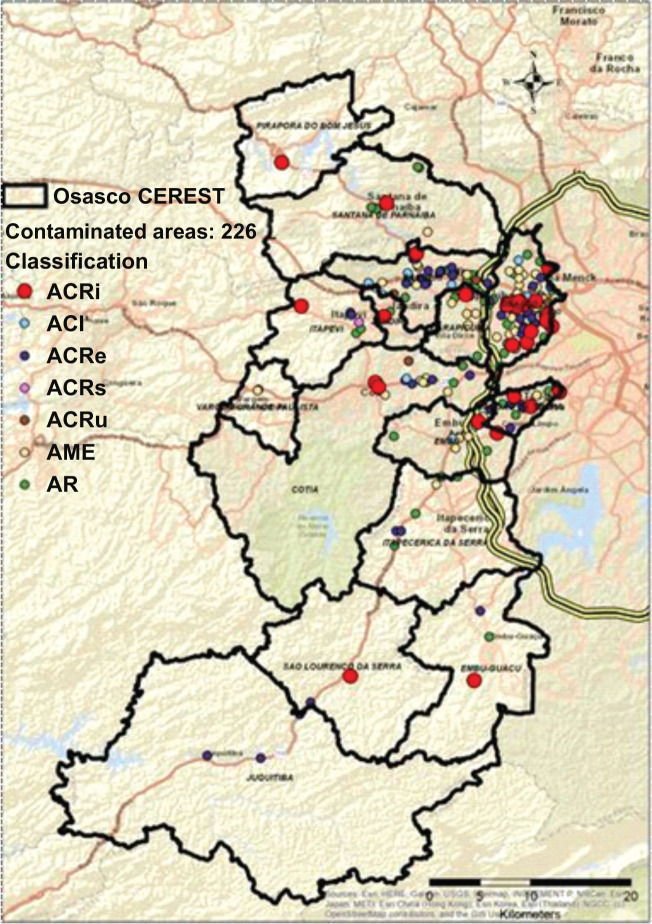
Map depicting all contaminated areas in the Osasco Occupational Health Reference Center (CEREST) region, 2017. ACI = contaminated area under investigation; ACRe = contaminated area in the process of restoration; ACRi = contaminated area with confirmed risk; ACRu = contaminated area in the process of reuse; AME = area in the process of monitoring for closure; AR = area rehabilitated for declared use; ACRs = critical contaminated area. Source: Digital Map of Municipalities and Highways: Brazilian Institute of Geography and Statistics (IBGE); Contaminated Areas: São Paulo State Environmental Company (CETESB), 2017.

The most concerning areas, requiring immediate action are ACRi, as they present the greatest potential for damage to the health of exposed workers and the general population. Among all the contaminated areas CETESB reported in 2017, 226 contaminated areas were identified; and 28 were ACRi, as shown on the map in Figure 1.

The municipalities with the highest concentrations of contaminated areas, considering all classifications, including the ACRi, are Osasco, Taboão da Serra, and Barueri, where are also the main access roads to the Osasco CEREST region, such as the Rodoanel, the Raposo Tavares highway, and the Castelo Branco highway, among others.

**Figure 2:**
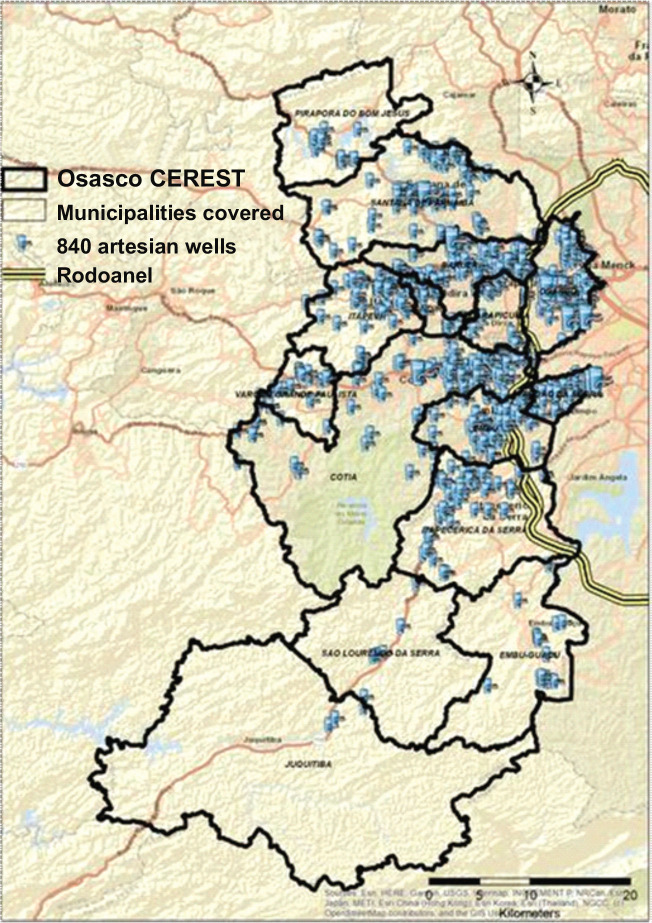
Map of potable artesian wells registered in the Osasco Occupational Health Reference Center (CEREST) region, 2019. Source: Digital Map of Municipalities and Roads: Brazilian Institute of Geography and Statistics (IBGE); Artesian Wells: Groundwater Information System (SIAGAS), 2019.

The artesian wells in the Osasco CEREST region were spatially located, totaling 840 wells registered with SIAGAS (Figure 2), which allowed the toxicity potential of these wells to be verified in relation to the areas contaminated by service stations.

Figure 2 shows a map of the concentration of Carapicuíba, and Barueri, where are also the main artesian wells in the most populated regions, such areas with confirmed contamination, especially the as the municipalities of Osasco, Taboão da Serra, working population.

**Figure 3:**
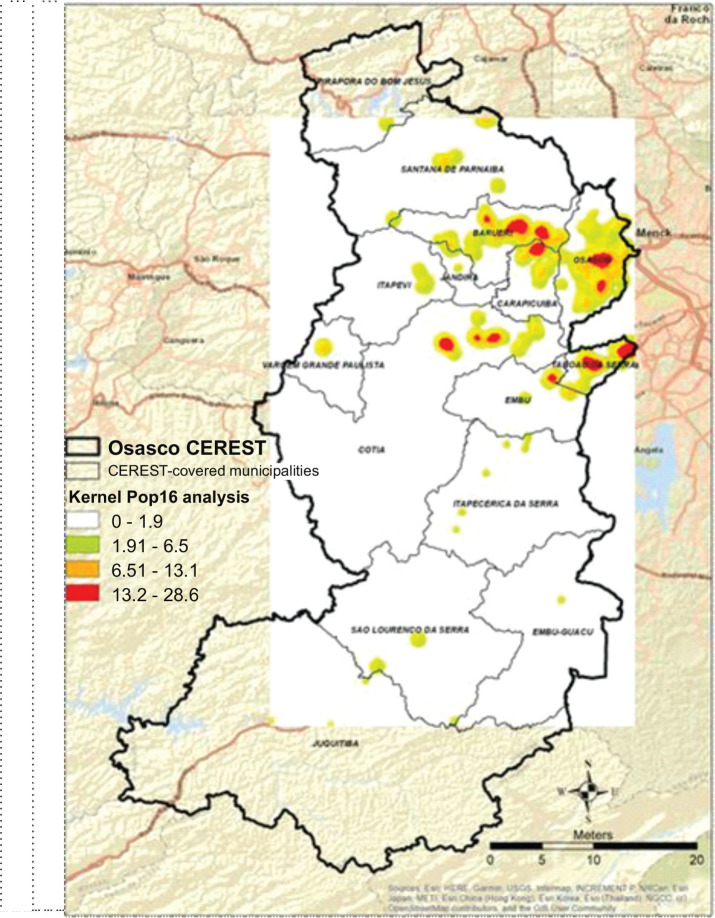
Kernel ratio map of the Osasco Occupational Health Reference Center (CEREST) region, 2017. Source: Digital Map of Municipalities and Roads: Brazilian Institute of Geography and Statistics (IBGE); 2016 Population: IBGE; Contaminated Areas: São Paulo State Environmental Company (CETESB), 2017.

The highest concentrations of these areas for the Kernel ratio of all contaminated areas, based on populations at risk were located through analysis of the 2016 population estimate (Figure 3).

According to the Kernel ratio map shown in Figure 3, a higher heat intensity can be seen in the municipalities of Osasco, Carapicuíba, Barueri, Taboão da Serra, and to the north of the municipality of Cotia, demonstrating a greater risk for the population exposed to contaminated areas in these locations, especially confirmed risk areas.

**Figure 4:**
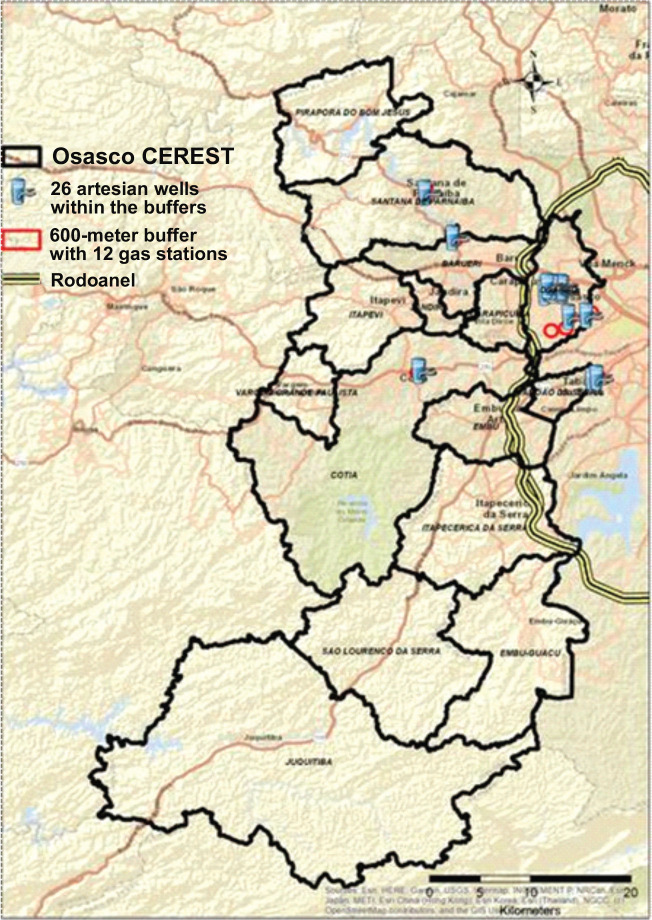
Map of potable artesian wells located within the 600-meter buffer of contaminated areas with confirmed risk (ACRIs) by service stations in the region of the Osasco Occupational Health Reference Center (CEREST), 2017. Source: Digital Map of Municipalities and Highways: Brazilian Institute of Geography and Statistics (IBGE); Contaminated Areas: São Paulo State Environmental Company (CETESB), 2017; Artesian Wells: Groundwater Information System (SIAGAS), 2019.

**Figure 5:**
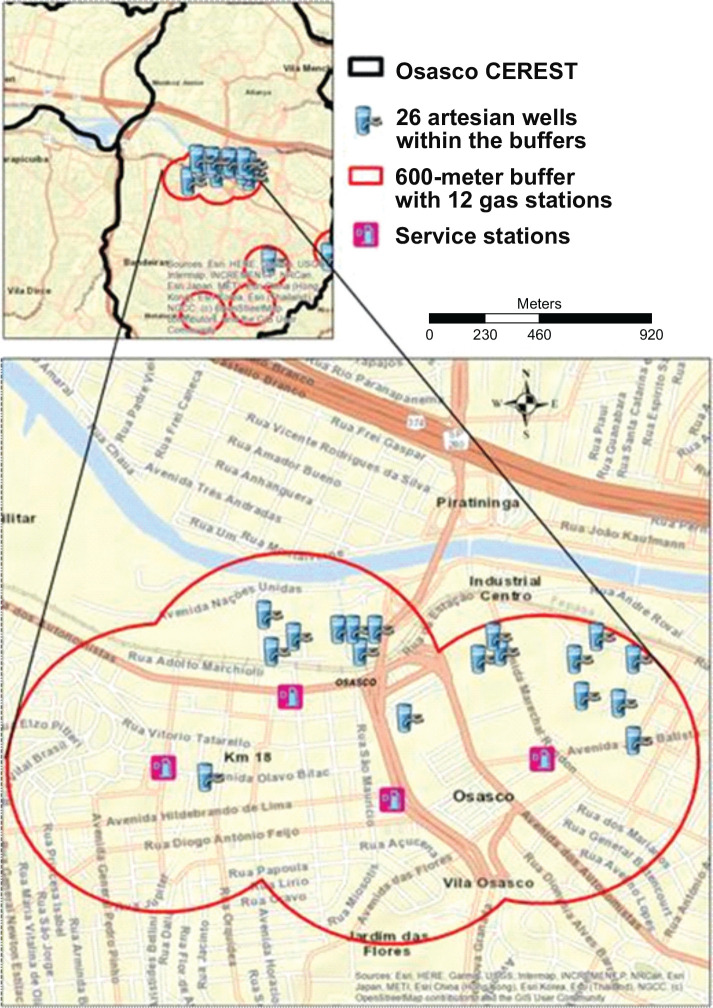
Map of potable artesian wells located within the 600-meter buffers of the contaminated areas with confirmed risk (ACRIs) by service stations in the municipality of Osasco, SP, 2017. The region with 69% of artesian wells within 600-meter buffers and service stations stands out. Source: Digital Map of Municipalities and Highways: Brazilian Institute of Geography and Statistics (IBGE); Contaminated Areas: São Paulo State Environmental Company (CETESB), 2017; Artesian Wells: Groundwater Information System (SIAGAS), 2019.

Figure 4 shows that of the 840 artesian wells located in the Osasco CEREST region, only 26 were within the 600-meter radius established from the 28 contaminated areas with confirmed risk in the region. In Osasco, Parnaíba, 1 artesian well each. Of the 28 ACRi, 12 are 20 artesian wells were found; in Taboão da Serra and service stations, with the highest concentration in the Cotia, 2 artesian wells; and in Barueri and Santana de municipality of Osasco, as shown in Figure 5.

## DISCUSSION

Since the Brazilian National Benzene Agreement was signed in 1994, Occupational Health Surveillance (VISAT) has been monitoring exposure to occupational health problems. However, population estimates of these exposures are scarce, both because of difficulties in accessing data sources and due to unavailable technologies.^11^

According to the Ministry of Labor Regulatory Standard 9, Environmental Risk Prevention Programs are required to measure the quantities of certain chemical agents in industries of public interest. However, this data is not available to VISAT or for scientific research.^11^

The Job Exposure Matrices are used worldwide to estimate the extent of exposure in work environments in large geographical areas, such as municipalities, states, or countries.^11^ Thus, for Brazil, potential exposure to benzene is estimated at more than 7 million workers, of which more than 770,000 were directly exposed to benzene in 2010. Among the occupational groups most exposed to benzene are service station workers and machine and engine operators and mechanics, as their activities involve handling benzene as a solvent, diluent, lubricant, or degreaser for cleaning parts and equipment.^11^

Among workers exposed to benzene, men are more exposed than women, and their exposure is more related to the occupational group of service station workers, while women are more likely to be exposed to benzene in the group of machine and engine operators. In terms of states, the highest prevalence of occupational exposure to benzene is found in the state of São Paulo. Mortality from leukemia among workers exposed to benzene is twice as high as among the general population.^11^

The population density in the RMSP, combined with a larger automobile fleet and a high concentration of service stations, especially in the Osasco CEREST region, leads to contamination and exposure of service station workers to benzene.

According to Silva & Barra,^18^ groundwater contamination with oil derivatives is extremely toxic to human health and makes the quality of water from artesian wells, which are also used by service stations to wash cars, unviable.

Technologies to restore confirmed risk areas contaminated by service stations and enable them to be used after being contaminated require huge costs for service station owners, ranging from R$514,450.00 to R$1,494,750.71 to complete restoration.^19^ Thus, after CETESB assessment confirming the contamination, the owners abandon the site instead of remediating the area, generating an environmental liability that causes a major social and public health problem.^19-21^

It is worth noting that this study has limitations when it comes to using a data source such as the Notifiable Diseases Information System, as this system reported only cases of acute contamination events related to exposure at work, rather than chronic events. Therefore, given the high possibility of underreporting, the data on notified workers (only 15 from 2010 to 2018, no cases in 2017, and even fewer cases when checking whether a Work Accident Report was issued) were not used in this study. It is also important to point out that, because this study uses an ecological design, causal relationships cannot be assessed.

This study has contributed to VISAT and also to the actions of the Osasco CEREST in caring for workers in the municipalities it covers, especially those exposed to contaminants from areas with confirmed risks. It also points to situations that alert some government bodies, such as the Brazilian National Water and Sanitation Agency (Agência Nacional de Águas e Saneamento Básico), to control the quality of water from groundwater for use in leisure, in the countryside, and for human consumption.

## CONCLUSIONS

Osasco, Cotia, Barueri, Taboão de Serra, and Santana de Parnaíba are the municipalities with ACRi that extract water from artesian wells with a high potential for benzene contamination.

There is an urgent need for better enforcement in areas with environmental liabilities, such as service stations that have failed to implement mitigation measures and do not have company insurance to manage the business risk. Long-term monitoring of workers exposed to benzene in the Osasco CEREST region is also essential.

Further population-based studies are needed to gather data on service station workers exposed to benzene, with a view to verifying the risk factors, their magnitude, and the causal relationships in this occupational category.
